# Supplementary material for: The Effect of Tropical Temperatures on the Quality of RNA Extracted from Stabilized Whole-Blood Samples
Source: Int J Mol Sci. 2022 Sep 13;23(18):10609. doi: 10.3390/ijms231810609 (PMC9503649; doi:10.3390/ijms231810609)
Supplement: Supplementary file 1 [file ijms-23-10609-s001.zip › ijms-1892099-supplementary.pdf]

Supplementary file 1: Supplementary Figures

Figure S1: Normalized total RNA yield, integrity and purity comparisons between RNA isolation protocols (silica-membrane column-based vs. magnetic beads) for PAXgene® and Tempus™ Blood RNA tubes. Bar graphs showing total RNA yields normalized to input whole blood volume, RNA Integrity Number (RIN), OD A260/280 and A260/230 ratios for silica-membrane column-based and magnetic beads based (MagMAX™) RNA purification protocols compatible with PAXgene® and Tempus™ blood collection tubes (matched n=3). Each bar represents the mean value, and the error bar indicates ± SE. Data from only two subjects were available on the Tempus™ collection system due to the unavailability of Tempus™ blood collection tubes from the same batch. The dashed lines at A260/A280 ratio >2.0 and A260/A230 ratio 2.0-2.2 represent the generally accepted range for high-quality RNA.

Ns, non-significant.

Figure S2: Reverse transcription at Full *vs.* Quarter concentration. The graph depicts RT-qPCR efficiency at reverse transcription using 5U (5units/µL; Quarter) reactions compared with 20U (Full) reactions (manufacturer's recommended protocol) for high-quality RNA (*i.e.*, freshly extracted) and low-quality RNA (*i.e.*, incubated 5 days at 40°C) extracted from the two RNA stabilization systems (PAXgene® and Tempus™). Unpaired t-test results determined no differences between Full *vs.* Quarter reaction concentrations on Fresh extractions while Full strength of RT was significantly efficient compared to the Quarter concentration of RT when evaluating low-quality, degraded RNA. Two-Way ANOVA analysis indicated a significant interaction between tube type and their condition/RNA quality (Fresh or 5 days at 40°C),

indicating that the relationships between Ct value and reverse transcription concentration depend on tube type and RNA quality. $^{**}p < 0.01$, $^{*}p < 0.05$, ns – non-significant.

Figure S3: qPCR inhibitory behaviour at serially diluted (log_2 dilutions) total RNA samples measured with SDHA (short primers) and differences between tube types. Whole blood extracted at Fresh and Frozen conditions from the two different tube types (PAXgene® and Tempus™) were tested to investigate if there were any lingering inhibitory factors from PAXgene® or Tempus™ tubes **A.** Ct results generated at Log-linear amplification-capable sample dilutions exhibit a straight line (beyond the dashed vertical line, at 30ng/uL), while inhibited dilutions form a curved line (before the dashed vertical line, at lower dilutions >30ng/uL). Tube type-based inhibition would be expected if present data shifted across the x-axis, with inhibitory factors in tubes diluting out each titration, which is not observed here.

Figure S4: Cycle threshold (Ct) of the housekeeping gene, *SDHA* assessed for RNA samples extracted from PAXgene® and Tempus™ at 'Fresh' and 'Control' conditions. Box plots shows Ct values for *SDHA* with100-200 base pair (bp) (short-amplicon) for Fresh *vs.* Day1/Control; immediately frozen at -80°C for later extraction (matched n=6). Paired t-test results determined no overall differences between Fresh *vs.* Control conditions on Ct values.

Figure S5: Cycle thresholds (Ct) in dependence on amplicon length and RNA integrity (RIN) for *TBP* and *18*s. The scatter plots show the Ct values in dependence on amplicon

length and RNA integrity (RIN) for different amplicon lengths of *TBP* and *18s* when extracted from PAXgene® and Tempus™ at different storage lengths.

Figure S6: A260/230 ratios for PAXgene® and Tempus™ tubes blood collection systems under suboptimal tropical storage conditions. A260/A230 ratios determined spectrophotometrically across the different conditions [Fresh (n=8), Day1/Control; immediately frozen at -80°C for later extraction and various temperatures (25, 30, 35, 40°C) (matched subjects n=3)]. The dashed line indicates OD260/A230 = 2.0 for high-quality RNA.

Supplementary file 2: Supplementary Tables

Table S1: Summarised technical characteristics of RNA extraction methods from PAXgene® and Tempus™ Blood RNA Tubes.

Blood stabilization tube type	**PAXgene® Blood RNA Tubes**		**Tempus™ Blood RNA tubes**	
RNA isolation protocol	PAXgene® Blood RNA Kit	MagMAX™ for Stabilized Blood Tubes RNA Isolation Kit, compatible with PAXgene® Blood RNA tubes	Tempus™ Spin RNA Isolation Kit	MagMAX™ for Stabilized Blood Tubes RNA Isolation Kit, compatible with Tempus™ Blood RNA tubes
Blood volume	2.5mL	2.5mL	3mL	3mL
RNA isolation method	spin columns	magnetic beads	spin columns	magnetic beads
RNA processing method	manual	manual	manual	manual
Proteinase K treatment requirement	yes	yes	no	yes
DNase digestion implemented in Kit	yes	yes	no (optional)	yes
Elution volume (µL)	40	20-80	90	20-80
Expected RNA yield	>3µg	3-18µg	6-25µg	3-25µg

Table S2: List of primers used in this study for quantitative real-time PCR and their efficiencies.

Regression line fit to raw Cq values against log2 diluted cDNA concentrations. R^2 values were calculated using Pearson's correlation.

Efficiencies were calculated as per MIQE guidelines (E = 10^((-1/slope)-1)) using the slope of the regression line previously described.

Gene	GenBank Accession	Primer Bank ID	Primer Sequence	Amplicon Length (bp)	Standard Curve linear equation	R^2	Efficiency (%)
TBP (Short)	NM_003194	285026518c1	**Forward** CCACTCACAGACTCTCACAAC **Reverse** CTGCGGTACAATCCCAGAACT	127	y = -3.203x + 23.385	0.995	105.21
TBP (Mid)	NM_003194	285026518c2	**Forward** CCACTCACAGACTCTCACAAC **Reverse** AATCAGTGCCGTGGTTCGTG	274	y = -3.0639x + 24.876	0.984	112.02
SDHA (Short)	NM_004168	156416002c1	**Forward** CAAACAGGAACCCGAGGTTTT **Reverse** CAGCTTGGTAACACATGCTGTAT	201	y = -3.2634x + 20.886	0.977	102.46
SDHA (Mid)	NM_004168	156416002c2	**Forward** CAAACAGGAACCCGAGGTTTT **Reverse** GGTGTCGTAGAAATGCCACCT	301	y = -3.4536x + 22.792	0.993	94.78
18s (Short)	NM_022551	14165467c1	**Forward** GCGGCGGAAAATAGCCTTTG **Reverse** GATCACACGTTCCACCTCATC	139	y = -3.3503x + 17.163	0.996	98.83
18s (Mid)	NM_022551	14165467c2	**Forward** GCGGCGGAAAATAGCCTTTG **Reverse** GACCTGGCTGTATTTTCCATCC	229	y = -3.3656x + 17.715	0.993	98.21

Table S3: Multiple linear regression model outputs for RNA concentration, A260/A280 and RIN values at each temperature condition.

RNA concentration (ng/µL) - Spectrophotometer				
**25°C (adj-R^2 = 0.832 (p = 3.494e-05)**				
	Estimate	Std. Error	Statistic	p value
(Intercept)	73.893	16.145	4.577	0.0006***
Tube type: Tempus	98.962	13.182	7.507	7.16e-06***
Days7	0.988	16.145	0.061	0.952
Days10	-15.822	16.145	-0.980	0.346
Subject B	-31.642	16.145	-1.960	0.074
Subject C	57.528	16.145	3.563	0.003900**
**30°C (adj-R^2 = 0.761 (p = 0.0003)**				
(Intercept)	50.483	17.482	2.888	0.014*
Tube type: Tempus	69.487	14.274	4.868	0.0004***
Days7	-0.655	17.482	-0.037	0.971
Days10	3.330	17.482	0.190	0.852
Subject B	-34.077	17.482	-1.949	0.075
Subject C	67.992	17.482	3.889	0.002**
**35°C (adj-R^2 = 0.484 (p = 0.020)**				
(Intercept)	79.980	23.110	3.460	0.005***
Tube type: Tempus	45.300	18.870	2.401	0.033*
Days7	-25.120	23.110	-1.087	0.298
Days10	-39.210	23.110	-1.696	0.116
Subject B	-30.830	23.110	-1.334	0.207
Subject C	49.340	23.110	2.135	0.054
**40°C (adj-R^2 = 0.630 (p = 0.003)**				
(Intercept)	74.480	13.910	5.355	0.0002***
Tube type: Tempus	29.530	11.360	2.600	0.023*
Days7	-29.340	13.910	-2.109	0.057
Days10	-19.930	13.910	-1.433	0.177
Subject B	-38.470	13.910	-2.766	0.017*
Subject C	27.260	13.910	1.960	0.074
**A260/A280**				
**25°C (adj-R^2 =-0.186 (p = 0.794)**				
	Estimate	Std.error	Statistic	p value
(Intercept)	2.196	0.022	101.726	<2e-16***
Tube type: Tempus	-0.018	0.018	-1.020	0.328
Days7	-0.006	0.022	-0.294	0.774
Days10	-0.006	0.022	-0.263	0.797
Subject B	0.006	0.022	0.271	0.791
Subject C	-0.017	0.022	-0.781	0.450

**30˚C (adj-R^2 = 0.469 (*p* = 0.023)**				
(Intercept)	2.204	0.020	111.518	<2e-16***
Tube type: Tempus	-0.053	0.016	-3.277	0.007**
Days7	-0.010	0.020	-0.506	0.622
Days10	-0.024	0.020	-1.197	0.254
Subject B	-0.054	0.020	-2.732	0.018*
Subject C	-0.017	0.020	-0.856	0.409
**35˚C (adj-R^2 = 0.372 (*p* = 0.055)**				
(Intercept)	2.189	0.029	74.899	<2e-16***
Tube type: Tempus	-0.072	0.024	-3.022	0.011*
Days7	-0.026	0.029	-0.898	0.387
Days10	-0.018	0.029	-0.605	0.557
Subject B	-0.056	0.029	-1.916	0.079
Subject C	0.002	0.029	0.071	0.944
**40˚C (adj-R^2 = 0.706 (*p* = 0.001)**				
(Intercept)	2.172	0.018	117.830	<2e-16***
Tube type: Tempus	-0.093	0.015	-6.157	4.89e-05***
Days7	-0.018	0.018	-0.968	0.352
Days10	-0.027	0.018	-1.469	0.167
Subject B	-0.020	0.018	-1.058	0.311
Subject C	0.024	0.018	1.307	0.216
**$\log_2$(RIN)**				
**25˚C (adj-R^2 = 0.666 (*p* = 0.002)**				
	Estimate	Std.error	Statistic	*p* value
(Intercept)	2.608	0.081	32.135	5.21e-13***
Tube type: Tempus	0.018	0.066	0.276	0.787
Days7	-0.241	0.081	-2.967	0.012*
Days10	-0.483	0.081	-5.947	6.75e-05***
Subject B	-0.025	0.081	-0.303	0.767
Subject C	-0.142	0.081	-1.746	0.106
**30˚C (adj-R^2 = 0.744 (*p* = 0.0004)**				
(Intercept)	1.868	0.106	17.642	6e-10***
Tube type: Tempus	0.472	0.086	5.458	0.0001***
Days7	-0.111	0.106	-1.043	0.317
Days10	-0.474	0.106	-4.477	0.001***
Subject B	0.016	0.106	0.147	0.885
Subject C	-0.138	0.106	-1.306	0.216
**35˚C (adj-R^2 = 0.623 (*p* = 0.004)**				
(Intercept)	1.737	0.138	12.630	2.73e-08***
Tube type: Tempus	0.496	0.112	4.416	0.001***
Days7	-0.383	0.138	-2.783	0.017*
Days10	-0.437	0.138	-3.177	0.008**
Subject B	-0.113	0.138	-0.823	0.427

Subject C	-0.170	0.138	-1.239	0.239
**40°C (adj-R^2 = 0.579 (*p* = 0.01)**				
(Intercept)	1.500	0.063	23.676	1.93e-11***
Tube type: Tempus	0.045	0.052	0.875	0.399
Days7	-0.179	0.063	-2.822	0.015*
Days10	-0.317	0.063	-5.007	0.0003***
Subject B	0.023	0.063	0.355	0.729
Subject C	-0.072	0.063	-1.135	0.278

Table S4: Multiple linear regression model for RNA concentration measured by Agilent 2100 Bioanalyzer

**Agilent 2100 Bioanalyzer**	**RNA concentration (ng/µL)**			
**Explanatory variable**	Estimate	Std. Error	t value	*p* value
(Intercept)	57.627	214.498	0.269	0.789
Tube type: Tempus	438.154	128.773	3.403	0.001**
Days	14.905	27.193	0.548	0.586
Temperature	1.462	6.413	0.228	0.82
Tube type Tempus: Days	-7.112	9.09	-0.782	0.437
Tube type Tempus: Temperature	-7.8	3.341	-2.335	0.023*
Days: Temperature	-0.507	0.813	-0.624	0.535
Adjusted R-squared: 0.603 (*p* value: 9.591e-12)				
**25°C (adj-R^2 = 0.584 (*p* = 0.006)**				
	Estimate	Std.error	Statistic	*p* value
(Intercept)	163.830	51.120	3.205	0.008***
Tube type: Tempus	182.220	41.740	4.366	0.001***
Days7	41.830	51.120	0.818	0.429
Days10	-15.670	51.120	-0.306	0.765
Subject B	-111.670	51.120	-2.184	0.050*
Subject C	29.330	51.120	0.574	0.005**
**30°C (adj-R^2 = 0.724 (*p* = 0.001)**				
(Intercept)	79.170	42.050	1.882	0.084
Tube type: Tempus	180.330	34.340	5.252	0.0002***
Days7	-34.170	42.050	-0.812	0.432
Days10	26.670	42.050	0.634	0.538
Subject B	-72.830	42.050	-1.732	0.109
Subject C	112.830	42.050	2.683	0.020
**35°C (adj-R^2 = 0.451 (*p* = 0.027)**				
(Intercept)	68.280	48.030	1.421	0.181
Tube type: Tempus	82.670	39.220	2.108	0.057
Days7	22.000	48.030	0.458	0.655

Days10	-82.330	48.030	-1.714	0.112
Subject B	22.000	48.030	0.458	0.655
Subject C	136.170	48.030	2.835	0.015*
**40˚C (adj-R^2 = 0.596 (*p* = 0.005)**				
(Intercept)	86.000	35.557	2.419	0.032*
Tube type: Tempus	84.778	29.032	2.920	0.013*
Days7	-3.833	35.557	-0.108	0.916
Days10	-22.833	35.557	-0.642	0.533
Subject B	-38.167	35.557	-1.073	0.304
Subject C	118.500	35.557	3.333	0.006**

*** $p < 0.001$, ** $p < 0.01$, * $p < 0.05$ from separate multiple linear regression models with total RNA and RIN values as dependent variables and tube type, day and subjects as the independent variables for each temperature point.

Table S6: Two-way ANOVA results with multiple comparisons outputs for control and test conditions on PAXgene® and Tempus™ tube types on Ct values obtained for 18s, SDHA and TBP. **** p< 0.0001, *** p< 0.001, **p< 0.01, *p< 0.05, ns – non-significant

Bonferroni's multiple comparisons test	Predicted (LS) mean diff.	95.00% CI of diff.	Below threshold?	Summary	Adjusted *p* value
18s short (100-200bp)<br>PAXgene®					
25 - 1 vs. 25 - 5	0.216	-1.394 to 1.826	No	ns	>0.999
25 - 1 vs. 25 - 7	0.455	-1.155 to 2.065	No	ns	>0.999
25 - 1 vs. 25 - 10	0.303	-1.307 to 1.913	No	ns	>0.999
25 - 1 vs. 30 - 5	0.734	-0.876 to 2.344	No	ns	>0.999
25 - 1 vs. 30 - 7	-0.058	-1.668 to 1.552	No	ns	>0.999
25 - 1 vs. 30 - 10	-0.600	-2.210 to 1.010	No	ns	>0.999
25 - 1 vs. 35 - 5	0.038	-1.572 to 1.648	No	ns	>0.999
25 - 1 vs. 35 - 7	-0.384	-1.994 to 1.226	No	ns	>0.999
25 - 1 vs. 35 - 10	-0.088	-1.698 to 1.522	No	ns	>0.999
25 - 1 vs. 40- 5	-0.087	-1.697 to 1.523	No	ns	>0.999
25 - 1 vs. 40 - 7	-0.626	-2.236 to 0.984	No	ns	>0.999
25 - 1 vs. 40 - 10	-1.678	-3.288 to -0.068	Yes	*	0.035
Tempus™					
25 - 1 vs. 25 - 5	0.497	-1.113 to 2.107	No	ns	>0.999
25 - 1 vs. 25 - 7	0.371	-1.239 to 1.981	No	ns	>0.999
25 - 1 vs. 25 - 10	0.354	-1.446 to 2.154	No	ns	>0.999
25 - 1 vs. 30 - 5	-0.589	-2.199 to 1.021	No	ns	>0.999
25 - 1 vs. 30 - 7	0.458	-1.152 to 2.068	No	ns	>0.999
25 - 1 vs. 30 - 10	0.503	-1.107 to 2.113	No	ns	>0.999
25 - 1 vs. 35 - 5	0.288	-1.322 to 1.898	No	ns	>0.999
25 - 1 vs. 35 - 7	0.017	-1.593 to 1.627	No	ns	>0.999
25 - 1 vs. 35 - 10	0.025	-1.585 to 1.635	No	ns	>0.999
25 - 1 vs. 40- 5	-0.825	-2.435 to 0.785	No	ns	>0.999
25 - 1 vs. 40 - 7	0.123	-1.487 to 1.733	No	ns	>0.999
25 - 1 vs. 40 - 10	-0.543	-2.153 to 1.067	No	ns	>0.999
18s medium (200-300bp)<br>PAXgene®					
25 - 1 vs. 25 - 5	-0.332	-3.247 to 2.583	No	ns	0.953
25 - 1 vs. 25 - 7	-0.059	-5.358 to 5.241	No	ns	>0.999
25 - 1 vs. 25 - 10	-0.220	-5.786 to 5.347	No	ns	>0.999
25 - 1 vs. 30 - 5	0.142	-5.013 to 5.297	No	ns	>0.999
25 - 1 vs. 30 - 7	-0.944	-8.888 to 6.999	No	ns	0.942
25 - 1 vs. 30 - 10	-1.627	-7.669 to 4.415	No	ns	0.489

25 - 1 vs. 35 - 5	-0.590	-10.91 to 9.726	No	ns	1.000
25 - 1 vs. 35 - 7	-1.093	-9.542 to 7.357	No	ns	0.917
25 - 1 vs. 35 - 10	-1.036	-13.51 to 11.44	No	ns	0.992
25 - 1 vs. 40- 5	-1.014	-10.91 to 8.883	No	ns	0.972
25 - 1 vs. 40 - 7	-1.567	-6.057 to 2.924	No	ns	0.336
25 - 1 vs. 40 - 10	-3.090	-7.650 to 1.470	No	ns	0.105
Tempus™					
25 - 1 vs. 25 - 5	0.575	-3.909 to 5.060	No	ns	0.919
25 - 1 vs. 25 - 7	0.458	-4.390 to 5.306	No	ns	0.983
25 - 1 vs. 25 - 10	0.115	-80.75 to 80.98	No	ns	>0.999
25 - 1 vs. 30 - 5	0.169	-10.41 to 10.75	No	ns	>0.999
25 - 1 vs. 30 - 7	0.397	-8.594 to 9.389	No	ns	>0.999
25 - 1 vs. 30 - 10	0.284	-2.694 to 3.262	No	ns	0.982
25 - 1 vs. 35 - 5	-0.646	-14.10 to 12.81	No	ns	>0.999
25 - 1 vs. 35 - 7	-0.086	-7.871 to 7.699	No	ns	>0.999
25 - 1 vs. 35 - 10	-0.130	-5.603 to 5.343	No	ns	>0.999
25 - 1 vs. 40- 5	-0.962	-18.28 to 16.36	No	ns	>0.999
25 - 1 vs. 40 - 7	-0.093	-5.292 to 5.107	No	ns	>0.9999
25 - 1 vs. 40 - 10	-0.979	-10.28 to 8.320	No	ns	0.968
SDHA short (100-200bp)<br>PAXgene®					
25 - 1 vs. 25 - 5	-2.491	-24.83 to 19.85	No	ns	>0.999
25 - 1 vs. 25 - 7	-2.014	-7.929 to 3.901	No	ns	0.412
25 - 1 vs. 25 - 10	-2.829	-7.956 to 2.298	No	ns	0.162
25 - 1 vs. 30 - 5	-2.863	-7.071 to 1.345	No	ns	0.107
25 - 1 vs. 30 - 7	-3.270	-6.294 to -0.246	Yes	*	0.043
25 - 1 vs. 30 - 10	-3.832	-4.927 to -2.738	Yes	**	0.004
25 - 1 vs. 35 - 5	-3.030	-5.341 to -0.718	Yes	*	0.029
25 - 1 vs. 35 - 7	-3.624	-6.454 to -0.794	Yes	*	0.031
25 - 1 vs. 35 - 10	-3.649	-9.402 to 2.104	No	ns	0.123
25 - 1 vs. 40- 5	-3.626	-7.994 to 0.7429	No	ns	0.072
25 - 1 vs. 40 - 7	-3.948	-4.529 to -3.367	Yes	**	0.001
25 - 1 vs. 40 - 10	-4.670	-6.432 to -2.908	Yes	**	0.007
Tempus™					
25 - 1 vs. 25 - 5	1.209	-9.472 to 11.890	No	ns	>0.999
25 - 1 vs. 25 - 7	0.926	-9.730 to 11.580	No	ns	>0.999
25 - 1 vs. 25 - 10	0.859	-14.830 to 16.540	No	ns	>0.999
25 - 1 vs. 30 - 5	1.503	-7.459 to 10.460	No	ns	>0.999
25 - 1 vs. 30 - 7	0.856	-9.513 to 11.230	No	ns	>0.999
25 - 1 vs. 30 - 10	0.850	-7.348 to 9.048	No	ns	>0.999
25 - 1 vs. 35 - 5	0.316	-3.551 to 4.184	No	ns	>0.999
25 - 1 vs. 35 - 7	-0.039	-8.257 to 8.179	No	ns	>0.9999
25 - 1 vs. 35 - 10	0.083	-65.390 to 65.560	No	ns	>0.9999

25 - 1 vs. 40- 5	-0.179	-7.152 to 6.794	No	ns	>0.9999
25 - 1 vs. 40 - 7	-0.120	-7.101 to 6.860	No	ns	>0.9999
25 - 1 vs. 40 - 10	-2.802	-37.880 to 32.280	No	ns	>0.9999
SDHA medium (200-300bp)<br>PAXgene®					
25 - 1 vs. 25 - 5	-3.096	-4.734 to -1.458	Yes	****	<0.0001
25 - 1 vs. 25 - 7	-2.592	-4.230 to -0.954	Yes	***	0.0002
25 - 1 vs. 25 - 10	-3.905	-5.543 to -2.267	Yes	****	<0.0001
25 - 1 vs. 30 - 5	-4.165	-5.803 to -2.527	Yes	****	<0.0001
25 - 1 vs. 30 - 7	-4.810	-6.448 to -3.172	Yes	****	<0.0001
25 - 1 vs. 30 - 10	-5.814	-7.452 to -4.176	Yes	****	<0.0001
25 - 1 vs. 35 - 5	-4.893	-6.531 to -3.255	Yes	****	<0.0001
25 - 1 vs. 35 - 7	-5.387	-7.025 to -3.749	Yes	****	<0.0001
25 - 1 vs. 35 - 10	-6.029	-7.667 to -4.391	Yes	****	<0.0001
25 - 1 vs. 40- 5	-5.667	-7.305 to -4.029	Yes	****	<0.0001
25 - 1 vs. 40 - 7	-6.650	-8.288 to -5.012	Yes	****	<0.0001
25 - 1 vs. 40 - 10	-8.037	-9.675 to -6.399	Yes	****	<0.0001
Tempus™					
25 - 1 vs. 25 - 5	1.039	-0.599 to 2.677	No	ns	0.747
25 - 1 vs. 25 - 7	0.336	-1.302 to 1.974	No	ns	>0.999
25 - 1 vs. 25 - 10	0.526	-1.112 to 2.164	No	ns	>0.999
25 - 1 vs. 30 - 5	0.976	-0.662 to 2.614	No	ns	0.950
25 - 1 vs. 30 - 7	0.641	-0.997 to 2.279	No	ns	>0.999
25 - 1 vs. 30 - 10	0.103	-1.535 to 1.741	No	ns	>0.999
25 - 1 vs. 35 - 5	0.260	-1.378 to 1.898	No	ns	>0.999
25 - 1 vs. 35 - 7	-0.495	-2.133 to 1.143	No	ns	>0.999
25 - 1 vs. 35 - 10	-0.760	-2.587 to 1.066	No	ns	>0.999
25 - 1 vs. 40- 5	-0.601	-2.239 to 1.037	No	ns	>0.999
25 - 1 vs. 40 - 7	-0.851	-2.489 to 0.787	No	ns	>0.999
25 - 1 vs. 40 - 10	-2.941	-4.579 to -1.303	Yes	****	<0.0001
TBP short (100-200bp)<br>PAXgene®					
25 - 1 vs. 25 - 5	-2.338	-4.725 to 0.049	No	ns	0.059
25 - 1 vs. 25 - 7	-2.337	-4.724 to 0.050	No	ns	0.059
25 - 1 vs. 25 - 10	-2.657	-5.044 to -0.270	Yes	*	0.021
25 - 1 vs. 30 - 5	-2.931	-5.318 to -0.544	Yes	**	0.008
25 - 1 vs. 30 - 7	-3.110	-5.497 to -0.723	Yes	**	0.005
25 - 1 vs. 30 - 10	-3.350	-5.737 to -0.963	Yes	**	0.002
25 - 1 vs. 35 - 5	-2.859	-5.246 to -0.472	Yes	*	0.011
25 - 1 vs. 35 - 7	-3.328	-5.715 to -0.941	Yes	**	0.002
25 - 1 vs. 35 - 10	-3.027	-5.414 to -0.640	Yes	**	0.006
25 - 1 vs. 40- 5	-3.337	-5.724 to -0.950	Yes	**	0.002
25 - 1 vs. 40 - 7	-3.178	-5.565 to -0.791	Yes	**	0.004

25 - 1 vs. 40 - 10	-3.713	-6.100 to -1.326	Yes	***	0.001
Tempus™					
25 - 1 vs. 25 - 5	1.060	-1.327 to 3.447	No	ns	>0.999
25 - 1 vs. 25 - 7	0.506	-1.881 to 2.893	No	ns	>0.999
25 - 1 vs. 25 - 10	1.020	-1.367 to 3.407	No	ns	>0.999
25 - 1 vs. 30 - 5	1.338	-1.049 to 3.725	No	ns	>0.999
25 - 1 vs. 30 - 7	0.402	-1.985 to 2.789	No	ns	>0.999
25 - 1 vs. 30 - 10	0.264	-2.123 to 2.651	No	ns	>0.999
25 - 1 vs. 35 - 5	-0.084	-2.471 to 2.303	No	ns	>0.999
25 - 1 vs. 35 - 7	-0.172	-2.559 to 2.215	No	ns	>0.999
25 - 1 vs. 35 - 10	-1.934	-4.321 to 0.453	No	ns	0.204
25 - 1 vs. 40- 5	-0.251	-2.638 to 2.136	No	ns	>0.999
25 - 1 vs. 40 - 7	-0.096	-2.483 to 2.291	No	ns	>0.999
25 - 1 vs. 40 - 10	-0.796	-3.183 to 1.591	No	ns	>0.999
TBP medium (200-300bp)<br>PAXgene®					
25 - 1 vs. 25 - 5	-2.490	-5.256 to 0.277	No	ns	0.106
25 - 1 vs. 25 - 7	-2.597	-5.364 to 0.169	No	ns	0.080
25 - 1 vs. 25 - 10	-3.327	-6.094 to -0.560	Yes	*	0.010
25 - 1 vs. 30 - 5	-3.600	-6.367 to -0.833	Yes	**	0.005
25 - 1 vs. 30 - 7	-4.976	-7.743 to -2.209	Yes	****	<0.0001
25 - 1 vs. 30 - 10	-6.284	-9.050 to -3.517	Yes	****	<0.0001
25 - 1 vs. 35 - 5	-4.347	-7.114 to -1.581	Yes	***	0.001
25 - 1 vs. 35 - 7	-5.561	-8.328 to -2.795	Yes	****	<0.0001
25 - 1 vs. 35 - 10	-5.537	-8.303 to -2.770	Yes	****	<0.0001
25 - 1 vs. 40- 5	-5.139	-7.906 to -2.373	Yes	****	<0.0001
25 - 1 vs. 40 - 7	-5.217	-7.984 to -2.451	Yes	****	<0.0001
25 - 1 vs. 40 - 10	-5.859	-8.626 to -3.093	Yes	****	<0.0001
Tempus™					
25 - 1 vs. 25 - 5	1.211	-1.556 to 3.977	No	ns	>0.999
25 - 1 vs. 25 - 7	0.644	-2.123 to 3.411	No	ns	>0.999
25 - 1 vs. 25 - 10	0.910	-1.857 to 3.676	No	ns	>0.999
25 - 1 vs. 30 - 5	1.320	-1.447 to 4.086	No	ns	>0.999
25 - 1 vs. 30 - 7	0.311	-2.456 to 3.078	No	ns	>0.999
25 - 1 vs. 30 - 10	-0.068	-2.834 to 2.699	No	ns	>0.999
25 - 1 vs. 35 - 5	-0.067	-2.834 to 2.699	No	ns	>0.999
25 - 1 vs. 35 - 7	-0.635	-3.401 to 2.132	No	ns	>0.999
25 - 1 vs. 35 - 10	-3.655	-6.422 to -0.888	Yes	**	0.004
25 - 1 vs. 40- 5	-0.621	-3.388 to 2.146	No	ns	>0.999
25 - 1 vs. 40 - 7	-1.094	-3.861 to 1.672	No	ns	>0.999
25 - 1 vs. 40 - 10	-2.440	-5.207 to 0.326	No	ns	0.121
