# Supplementary figures and images for: The Effect of Tropical Temperatures on the Quality of RNA Extracted from Stabilized Whole-Blood Samples
Source: Int J Mol Sci. 2022 Sep 13;23(18):10609. doi: 10.3390/ijms231810609 (PMC9503649; doi:10.3390/ijms231810609)

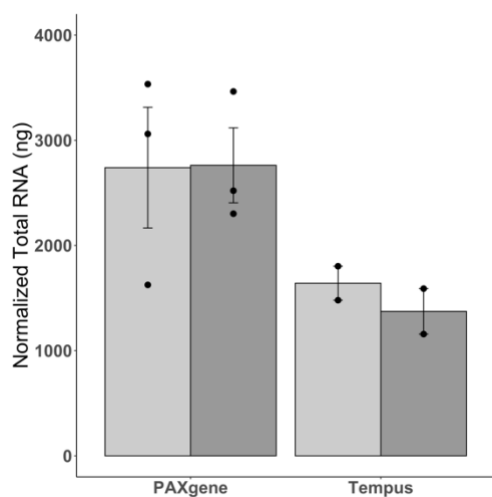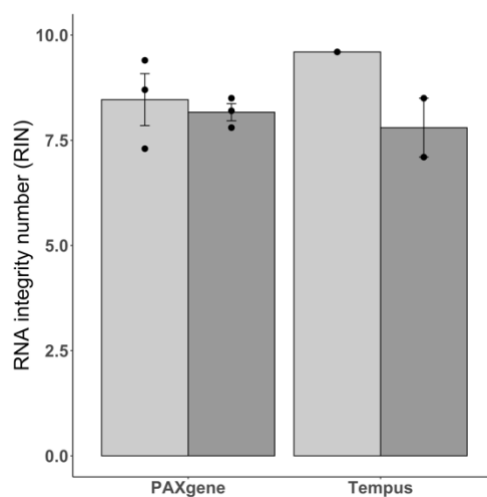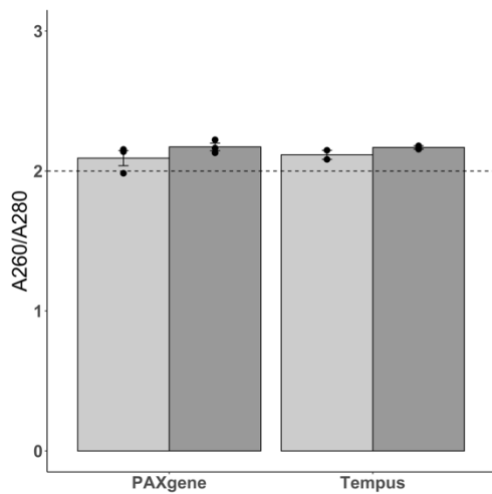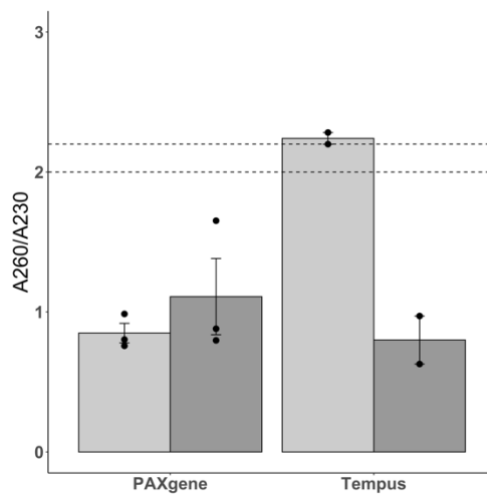

Method  Column  Magmax

Supplement: Supplementary file 1 [file ijms-23-10609-s001.zip › Figure S1.pdf]

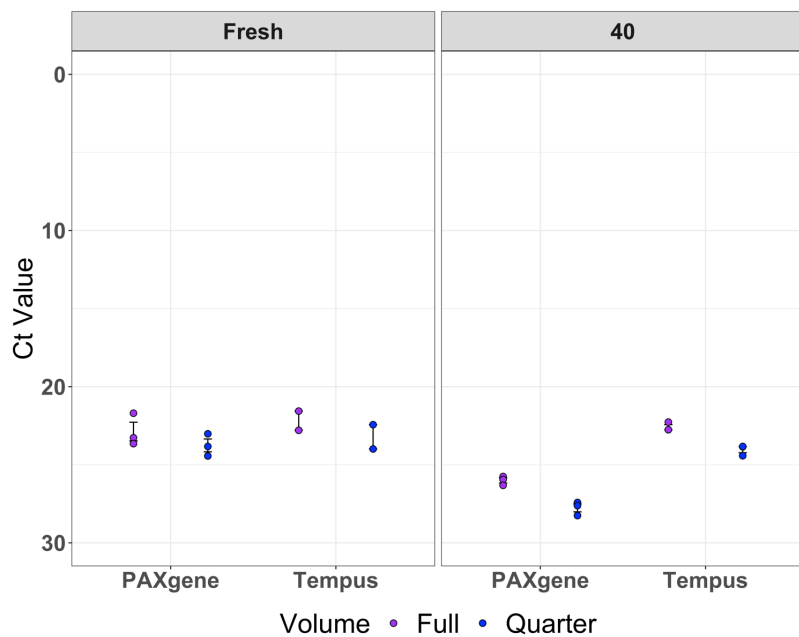

$$p_{\text{Volume: Condition}} = 0.252$$

$$p_{\text{Volume: Tube type}} = 0.841$$

$$p_{\text{Condition: Tube type}} = 8.52\text{e-}05$$

Supplement: Supplementary file 1 [file ijms-23-10609-s001.zip › Figure S2.pdf]

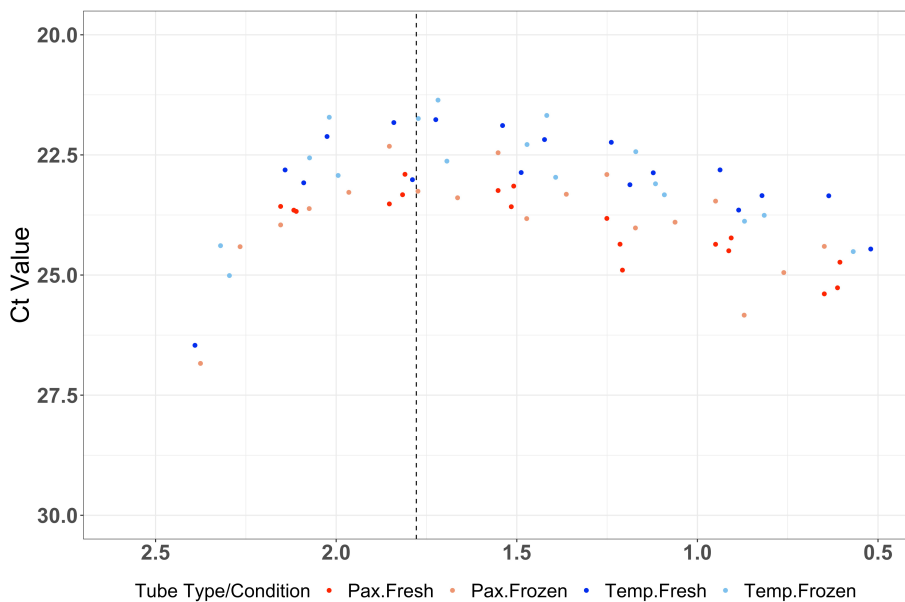

Supplement: Supplementary file 1 [file ijms-23-10609-s001.zip › Figure S3.pdf]

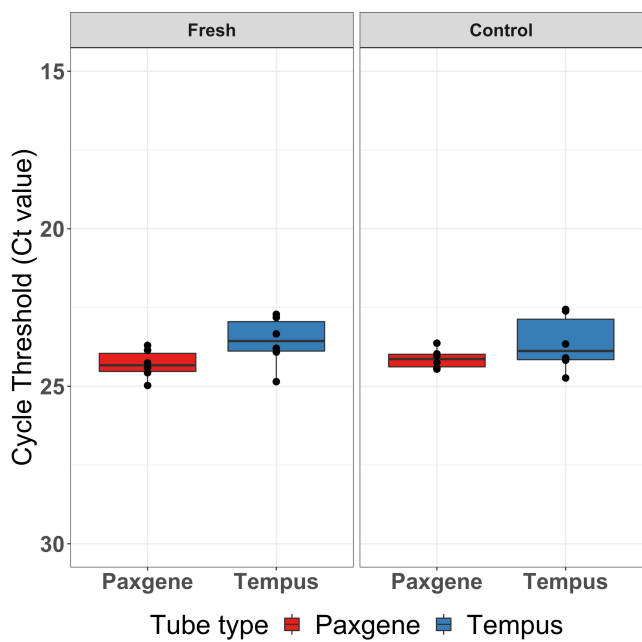

Supplement: Supplementary file 1 [file ijms-23-10609-s001.zip › Figure S4.pdf]

TBP (100-200 bp)

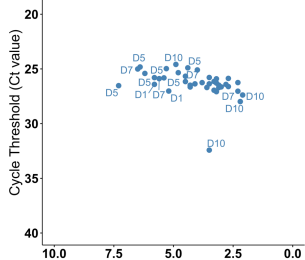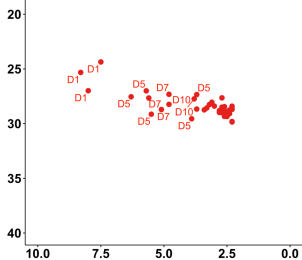

TBP (200-300 bp)

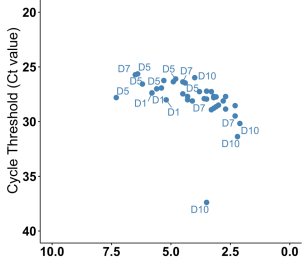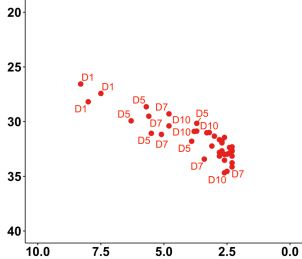

TBP (>300 bp)

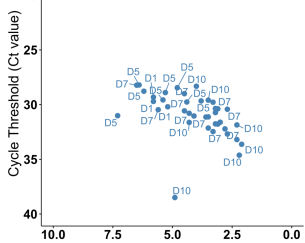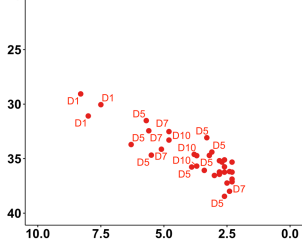

18s (100-200 bp)

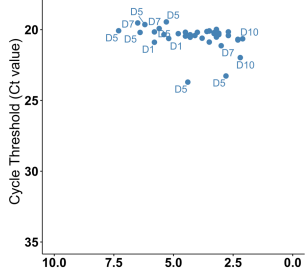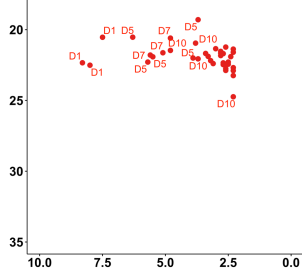

18S (200-300 bp)

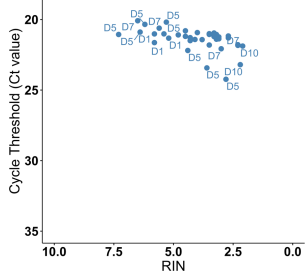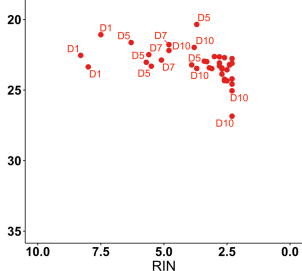

Tube type ● PAXgene ● Tempus

Supplement: Supplementary file 1 [file ijms-23-10609-s001.zip › Figure S5.pdf]

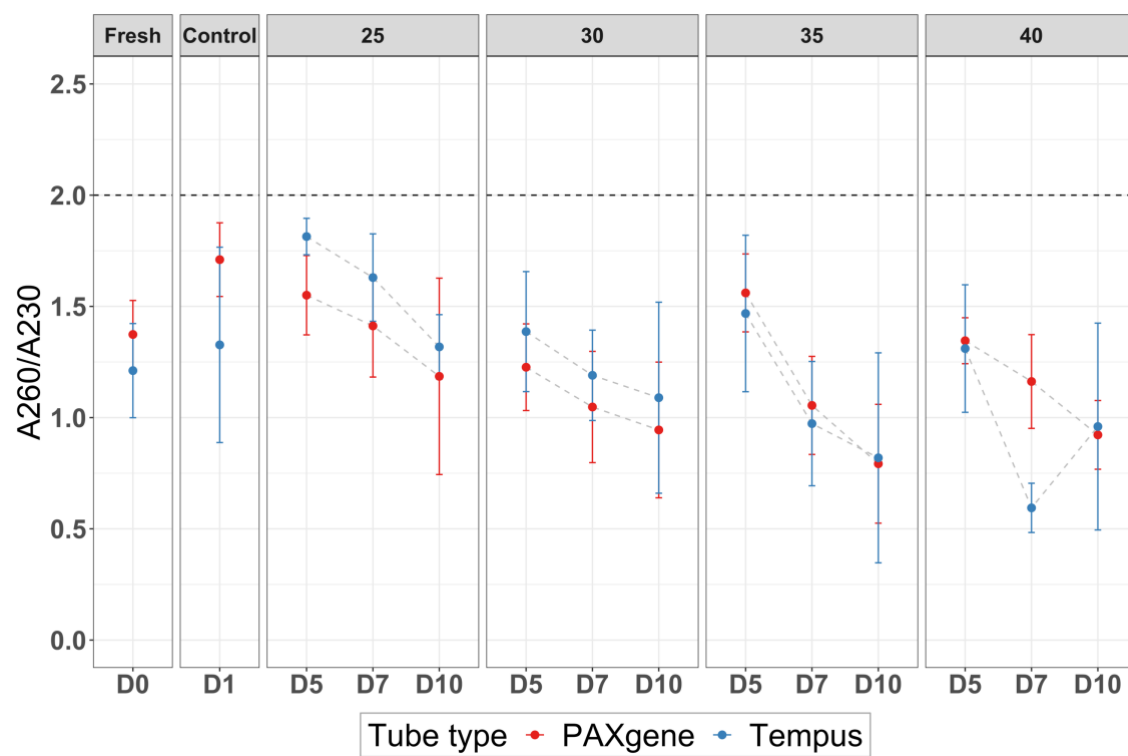

Supplement: Supplementary file 1 [file ijms-23-10609-s001.zip › Figure S6.pdf]
